# Association of maternal psychological distress and the use of childcare facilities with children's behavioral problems: the Tohoku Medical Megabank Project Birth and Three-Generation Cohort Study

**DOI:** 10.1186/s12888-022-04330-2

**Published:** 2022-11-11

**Authors:** Ippei Takahashi, Keiko Murakami, Mika Kobayashi, Saya Kikuchi, Ayaka Igarashi, Taku Obara, Mami Ishikuro, Fumihiko Ueno, Aoi Noda, Tomomi Onuma, Fumiko Matsuzaki, Natsuko Kobayashi, Hirotaka Hamada, Noriyuki Iwama, Masatoshi Saito, Junichi Sugawara, Hiroaki Tomita, Nobuo Yaegashi, Shigeo Kure, Shinichi Kuriyama

**Affiliations:** 1 Tohoku University Graduate School of Medicine, Sendai, Japan; 2 Tohoku Medical Megabank Organization, Tohoku University, 2-1 Seiryo-Machi, Aoba-Ku, Sendai, 980-8573 Japan; 3 Department of Psychiatry, Tohoku University Hospital, Sendai, Japan; 4 Tohoku University School of Medicine, Sendai, Japan; 5 Department of Pharmaceutical Sciences, Tohoku University Hospital, Sendai, Japan; 6 Department of Obstetrics and Gynecology, Tohoku University Hospital, Sendai, Japan; 7 International Research Institute of Disaster Science, Tohoku University, Sendai, Japan

**Keywords:** Prenatal psychological distress, Postnatal psychological distress behavioral problems, Childcare; childcare facility

## Abstract

**Background:**

Childcare facilities are a factor that lowers the established association of mother’s postnatal psychiatric symptoms with children's behavioral problems. However, no studies have considered the prenatal psychiatric symptoms yet. This study examined whether the use of childcare facilities moderates the association of maternal psychological distress in early pregnancy and at two years postpartum with behavioral problems in children aged four years.

**Methods:**

The present study was based on the data from 23,130 mother–child pairs participating in the Tohoku Medical Megabank Project Birth and Three-Generation Cohort Study. K6 was used to classify maternal psychological distress in early pregnancy and at two years postpartum into four categories: none in both prenatal and postnatal periods (none), only the prenatal period (prenatal only); only the postnatal period (postnatal only); both prenatal and postnatal periods (both). The children's behavioral problems were assessed using the Child Behavior Checklist for Ages 1½–5 (CBCL) aged four years. The clinical range of the externalizing, internalizing, and total problem scales of the CBCL was defined as having behavioral problems. To examine whether availing childcare facilities moderates the association between maternal psychological distress and children's behavioral problems, we conducted a stratified analysis based on the use of childcare facilities or not, at two years of age. The interaction term between maternal psychological distress and use of childcare facilities was included as a covariate in the multivariate logistic regression analysis to confirm the *p*-value for the interaction.

**Results:**

The prevalence of the clinical ranges of externalizing problems, internalizing problems, and clinical range of total problems were 13.7%, 15.4%, and 5.8%, respectively. The association of maternal psychological distress with a high risk of children's behavioral problems was significant; however, the association between prenatal only psychological distress and externalizing problems in the group that did not use childcare facilities was not significant. Interactions between the use of childcare facilities and maternal psychological distress on behavioral problems in children were not significant.

**Conclusions:**

Use of childcare facilities did not moderate the association of maternal psychological distress in early pregnancy and at two years postpartum with behavioral problems in children aged four years.

**Supplementary Information:**

The online version contains supplementary material available at 10.1186/s12888-022-04330-2.

## Introduction

Mental disorders are a severe health problem worldwide [1]. The American Psychological Association (APA) has defined the symptoms of depression as "not just sadness, but also a lack of interest and pleasure in daily activities, significant weight loss or gain, insomnia or excessive sleeping, lack of energy, inability to concentrate, feelings of worthlessness or excessive guilt and recurrent thoughts of death or suicide” [2]. Symptoms of anxiety has been defined as " feelings of tension, worried thoughts and physical changes like increased blood pressure” [3]. People with anxiety usually have recurring intrusive thoughts and worry, may avoid certain situations out of worry, and may also have physical symptoms such as sweating, trembling, dizziness, and a rapid heartbeat [3]. Several studies have ascertained that maternal psychiatric symptoms such as depression and anxiety during the prenatal or postnatal periods are associated with behavioral problems in children [4–14]. Therefore, the incidence of maternal psychiatric symptoms is a serious problem which adversely affects both the mother and the child.

Identifying the factors that moderate the association between maternal psychiatric symptoms and the risk of behavioral problems in children is crucial for both the children's development and maternal mental health. To the best of our knowledge, three studies have shown that childcare institutions, such as daycare, nursery, or kindergarten alleviate the association between maternal postpartum depressive symptoms and the risk of children's behavioral problems [11, 15, 16]. A study showed that parenting by other caregivers besides the mother reduced the harmful effects of maternal postpartum depression on the child [15]. One study showed a significant association with a lower risk of emotional problems among children who received group care compared to those who received care from their mothers in a group of children with their respective mothers experiencing persistent severe postpartum depressive symptoms [16]. Another study showed that the association between maternal postpartum depressive symptoms and children's behavioral problems was alleviated by providing at least half a day of formal childcare per week, at two years of age [11].

While the use of childcare facilities has been shown to alleviate the association between postpartum depressive symptoms and behavioral problems in children [11, 15, 16], there are no studies that have considered the psychiatric symptoms experienced in the prenatal period. Postpartum psychiatric symptoms are associated with a risk of behavioral problems in children through low quality of parenting [17], while prenatal psychiatric symptoms are associated with behavioral problems by affecting the fetus in utero [9, 17–19]. Based on the difference of the pathways through which maternal psychiatric symptoms affect children, it is necessary to examine whether the use of childcare facilities moderates the association between behavioral problems in children and maternal psychiatric symptoms, in both the prenatal and postnatal periods.

Considering the above circumstances, the aim of this study was to examine whether the use of childcare facilities moderates the association of maternal psychological distress (includes some of the symptoms of depression and anxiety) in early pregnancy and at two years postpartum.

## Methods

### Study design and population

**Fig. 1:**
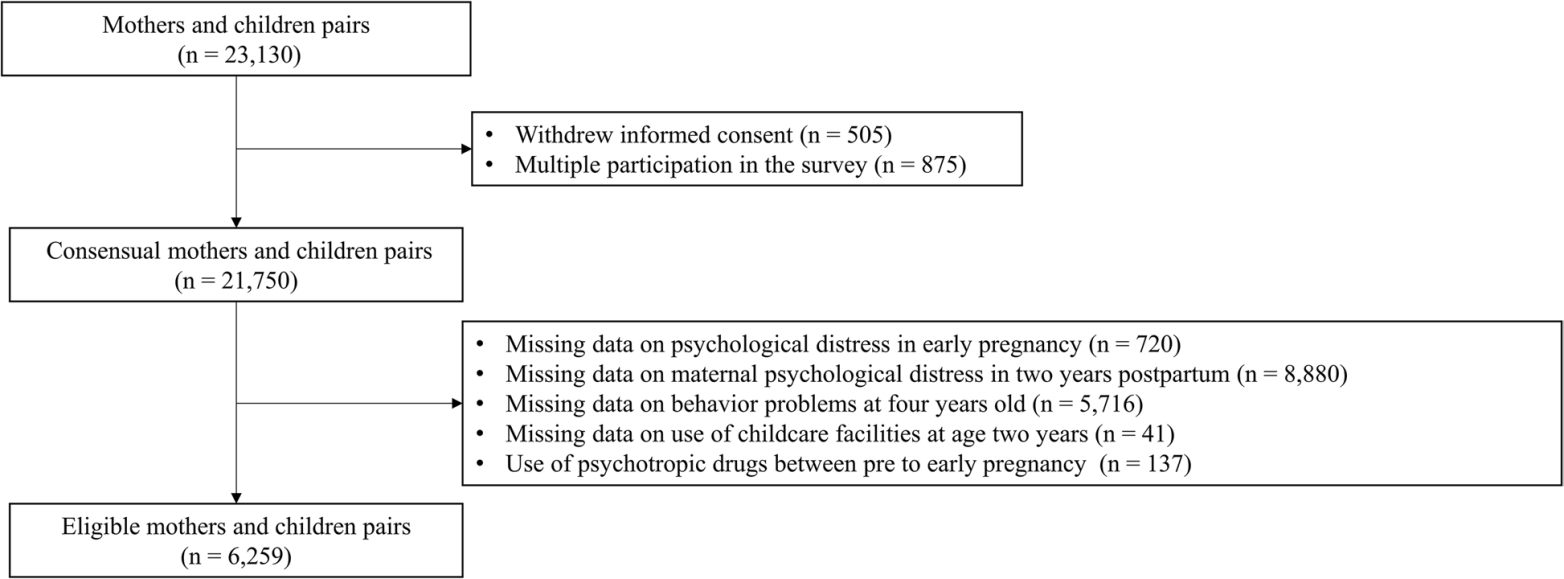
Flow chart of the participant exclusion criteria of this study

The present study was based on the Tohoku Medical Megabank Project Birth and Three Generation Cohort Study (TMM BirThree Cohort Study). The TMM BirThree Cohort Study details have been shown previously [20–23]. Between July 2013 and March 2017, the trained genome medical research coordinators explained the relevant information to the potential participants in each clinic, hospital, or community support center in Miyagi and Iwate prefectures, and obtained signed consent from each participant. Among the 32,986 pregnant women who were contacted, 23,406 pregnant women who had undergone multiple different pregnancies were registered. Furthermore, 23,130 mother and children pairs were evaluated as per the following study-appropriate exclusion criteria: withdrew informed consent (*n* = 505), multiple participation in the survey (*n* = 875), missing data on maternal psychological distress in early pregnancy (*n* = 720), missing data for maternal psychological distress at two years postpartum (*n* = 8,880), missing information on children's behavioral problems aged four years (*n* = 5,716), missing information on childcare facilities (*n* = 41), and use of psychotropic drugs between pre- to early pregnancy (*n* = 134). Finally, a total of 6,259 mother and child pairs were included in the analysis (Fig. 1).

### Maternal psychological distress

To assess the maternal psychological distress in early pregnancy (less than 14 weeks of pregnancy) and at two years postpartum, the Japanese version of the K6, which is a brief scale consisting of six questions was developed, validated, and used [24, 25]. We defined a K6 ≥ 5 points as psychological distress in this study. Prior research has proposed a K6 score of more than 5 as psychological distress [26, 27]. Furthermore, we classified mother and child pairs based on psychological distress into four categories: no psychological distress in both prenatal and postnatal periods (none), only the prenatal period (prenatal only), only the postnatal period (postnatal only), and both the prenatal and postnatal periods (both).

### Children's behavioral problems

The Child Behavior Checklist for Ages 1½–5 (CBCL) was used to assess the behavioral problems in children at the age of four years [28]. The CBCL is a parent-completed screening method, consisting of 100 items which is divided into seven syndrome scales (emotionally reactive, anxious/depressed, somatic complaints, withdrawn, sleep problems, attention problems, and aggressive behavior) [28, 29]. Furthermore, the scale of total problems is defined as the sum score of all 100 items on the CBCL, and the scale of internalizing problems is defined as the sum score of being emotionally reactive, anxious/depressed, having somatic complaints, and being withdrawn [28, 29]. The scale of externalizing problems is defined as the sum score of attention problems and aggressive behavior [28, 29]. The T-score (mean: 50, standard deviation: 10) for each of the scales was calculated and standardized for Japanese children [29]. A standardized T-score in the range of 60–63 indicates the borderline clinical range, while a standardized T-score of 64 or higher indicates the clinical range [29]. In this study, the clinical range (standardized T-score ≥ 64) of internalizing, externalizing, and total problems scales were analyzed as behavioral problems [29].

### Use of childcare facilities

Information on the use of childcare facilities was collected from the questionnaire at two years postpartum. The parents responded as to whether their children were attending childcare facilities at age two years or not.

### Covariates

We selected the covariates that may influence the association between maternal psychological distress and behavioral problems in children after reviewing the previous studies [4–14, 30–34]. We collected information on the child's sex from birth records. Information regarding maternal age (years) and parity (never, one, or more) was collected from the medical records. The maternal age was divided into four categories (< 25, 25–29, 30–34, ≥ 35 years). The information on maternal alcohol consumption in early pregnancy (never, ever, current), maternal cigarette smoking in early pregnancy (never, stopped before pregnancy, stopped after pregnancy, current), and paternal smoking in early pregnancy (never, stopped before pregnancy, stopped after pregnancy, current) were gathered from the questionnaire in early pregnancy [30–33]. Information on household income (< 4,000,000, 4,000,000–5,999,999, ≥ 6,000,000 Japanese yen/year) was collected from the questionnaire completed by the mothers during mid-pregnancy (14–27 weeks of pregnancy). The maternal educational attainment data (high school graduate or less, junior college or vocational college graduate, university graduate or above, others) were gathered a year after delivery [30, 34].

### Statistical analysis

The characteristics of the participants according to the four categories of maternal psychological distress have been described, and the *P*-values were determined from the chi-square test comparing the participants’ characteristics for the maternal psychological distress categories. Since all the variables were categorical, the data were presented as frequency and percentage. Furthermore, we compared the differences in characteristics between male and female children. To confirm the association between maternal psychological distress involving our categories and the scales of internalizing, externalizing, and total problems, we first conducted a multivariable logistic regression analysis, using the “none” category as a reference (no psychological distress in both prenatal and postnatal periods). To further examine whether the use of childcare facilities moderates the association between maternal psychological distress and children’s behavioral problems, we conducted a stratified analysis based on the use of childcare facilities at two years of age. Moreover, the interaction term between maternal psychological distress and the use of childcare facilities was included as a covariate in the multivariate logistic regression analysis to confirm the *P*-value for the interaction. Multiple imputations imputed incomplete confounders by the chained equation process [35]. Plausible synthetic values were generated from the given exposure, outcome, and other confounders in the data. Twenty sets of quasi-complete data were analyzed in the multivariate analyses independently and the estimates were integrated. All statistical analyses were performed using R, v.4.0.2 [36]. A *P*-value < 0.05 was considered as statistically significant.

## Results

### Characteristics of the study population

Table 1 shows the characteristics of the participants according to the four categories of maternal psychological distress. Of the 6,259 mothers that participated, 3,484 (55.7%) did not experience psychological distress in both the prenatal and postnatal periods; 1,055 (16.9%) experienced psychological distress in the prenatal period only, 701 (11.2%) experienced psychological distress in the postnatal period only, and 1,019 (16.3%) experienced psychological distress in both the prenatal and postnatal periods. Participants who experienced psychological distress in the prenatal or postpartum periods were characterized as younger, had no previous childbirth experience, and lower household income. Overall, 13.7% of children showed a clinical range of externalizing problems, 15.4% of the children showed a clinical range of internalizing problems, and 5.8% showed a clinical range of the total problems.

**Table 1:** Characteristics of participants with respect to maternal psychological distress

		Maternal psychological distress				
	Total	None	Prenatal only	Postnatal only	Both	
	(*n* = 6,259)	(*n* = 3,484)	(*n* = 1,055)	(*n* = 701)	(*n* = 1,019)	*P*-value^a^
	n (%)	n (%)	n (%)	n (%)	n (%)	
**Maternal age at delivery**						
< 25 (years)	291 (4.6)	134 (3.8)	56 (5.3)	31 (4.4)	70 (6.9)	< 0.001
25–30 (years)	1471 (23.5)	739 (21.2)	282 (26.7)	166 (23.7)	284 (27.9)	
30–35 (years)	2409 (38.5)	1313 (37.7)	411 (39.0)	288 (41.1)	397 (39.0)	
> 35 (years)	2088 (33.4)	1298 (37.3)	306 (29.0)	216 (30.8)	268 (26.3)	
**Parity, n (%)**						
Never	2949 (47.1)	1506 (43.2)	566 (53.6)	347 (49.5)	530 (52.0)	< 0.001
One or more	3294 (52.6)	1967 (56.5)	488 (46.3)	350 (49.9)	489 (48.0)	
Missing	16 (0.3)	11 (0.3)	1 (0.1)	4 (0.6)	0 (0.0)	
**Household income, n (%)**						
< 4,000,000 (JPY/year)	1976 (31.6)	994 (28.5)	341 (32.3)	239 (34.1)	402 (39.5)	< 0.001
4,000,000–6,000,000 (JPY/year)	2058 (32.9)	1154 (33.1)	373 (35.4)	210 (30.0)	321 (31.5)	
> 6,000,000 (JPY/year)	1959 (31.3)	1196 (34.3)	303 (28.7)	221 (31.5)	239 (23.5)	
Missing	266 (4.2)	140 (4.0)	38 (3.6)	31 (4.4)	57 (5.6)	
**Educational attainment, n (%)**						
High school graduate or less	1736 (27.7)	930 (26.7)	272 (25.8)	204 (29.1)	330 (32.4)	
Junior college or vocational college graduate	2288 (36.6)	1319 (37.9)	403 (38.2)	226 (32.2)	340 (33.4)	
University graduate or above	1825 (29.2)	1012 (29.0)	321 (30.4)	213 (30.4)	279 (27.4)	0.001
Others	10 (0.2)	9 (0.3)	0 (0.0)	1 (0.1)	0 (0.0)	
Missing	400 (6.4)	214 (6.1)	59 (5.6)	57 (8.1)	70 (6.9)	
**Maternal Alcohol drinking, n (%)**						
Never	2907 (46.4)	1612 (46.3)	492 (46.6)	325 (46.4)	478 (46.9)	0.912
Former	2092 (33.4)	1158 (33.2)	347 (32.9)	235 (33.5)	352 (34.5)	
Current	1246 (19.9)	706 (20.3)	212 (20.1)	140 (20.0)	188 (18.4)	
Missing	14 (0.2)	8 (0.2)	4 (0.4)	1 (0.1)	1 (0.1)	
**Maternal cigarette smoking, n (%)**						
Never	4081 (65.2)	2336 (67.0)	669 (63.4)	462 (65.9)	614 (60.3)	0.002
Stopped before pregnancy	1439 (23.0)	783 (22.5)	235 (22.3)	166 (23.7)	255 (25.0)	
Stopped after pregnancy	633 (10.1)	316 (9.1)	128 (12.1)	63 (9.0)	126 (12.4)	
Current	88 (1.4)	41 (1.2)	19 (1.8)	7 (1.0)	21 (2.1)	
Missing	18 (0.3)	8 (0.2)	4 (0.4)	3 (0.4)	3 (0.3)	
**Paternal cigarette smoking, n (%)**						
Never	1944 (31.1)	1057 (30.3)	327 (31.0)	239 (34.1)	321 (31.5)	0.212
Stopped before pregnancy	1518 (24.3)	863 (24.8)	264 (25.0)	170 (24.3)	221 (21.7)	
Stopped after pregnancy	168 (2.7)	85 (2.4)	31 (2.9)	24 (3.4)	28 (2.7)	
Current	2583 (41.3)	1458 (41.8)	423 (40.1)	264 (37.7)	438 (43.0)	
Missing	46 (0.7)	21 (0.6)	10 (0.9)	4 (0.6)	11 (1.1)	
**Child's sex, n (%)**						
Male	3251 (51.9)	1838 (52.8)	529 (50.1)	361 (51.5)	523 (51.3)	0.475
Female	3008 (48.1)	1646 (47.2)	526 (49.9)	340 (48.5)	496 (48.7)	
**Externalizing problems of CBCL, n (%)**						
Clinical range	859 (13.7)	314 (9.0)	144 (13.6)	146 (20.8)	255 (25.0)	< 0.001
**Internalizing problems of CBCL, n (%)**						
Clinical range	964 (15.4)	325 (9.3)	191 (18.1)	154 (22.0)	294 (28.9)	< 0.001
**Total problem of CBCL, n (%)**						
Clinical range	362 (5.8)	91 (2.6)	68 (6.4)	64 (9.1)	139 (13.6)	< 0.001
**Use child care facilities, n (%)**	2924 (46.7)	1645 (47.2)	463 (43.9)	328 (46.8)	488 (47.9)	< 0.001

*JPY* Japanese Yen, *CBCL* Child Behavior Checklist for Ages 1½–5

^a^Obtained using the chi-square test comparing participant’s characteristics for maternal psychological distress categories

Compared to the prevalence of behavioral problems in children, the maternal psychological distress in either the prenatal or postnatal period was higher than the incidence of no maternal psychological distress in both the prenatal and postnatal periods.

Additionally, the prevalence of behavioral problems was highest in the case of maternal psychological distress in both the prenatal and postnatal periods. The prevalence of behavioral problems in children was higher in mothers who experienced only postnatal psychological distress in comparison with mothers who experienced only prenatal psychological distress. The comparison of characteristics between male and female children showed no significant differences for all variables except for the externalizing problems. The prevalence of externalizing problems was higher in male children than the female children (Supplementary Table 1).

### Multivariable logistic regression analysis for maternal psychological distress and behavioral problems in children

Table 2 shows the odds ratios (ORs) and 95% CIs for the association of maternal psychological distress in early pregnancy and at two years postpartum with behavioral problems in children aged four years through a multivariate logistic regression analysis. On adjusting possible confounders, an association between the four categories of maternal psychological distress and the high risk of children's externalizing problems was found (adjusted OR[95% CI] = 1.55[1.25–1.92] for the prenatal only, 2.60[2.09–3.24] for the postnatal only, 3.17[2.63–3.83] for the both), internalizing problems (2.06[1.69–2.52] for the prenatal only, 2.68[2.16–3.34] for the postnatal only, 3.80[3.17–4.57] for the both) and total problems (2.39[1.73–3.31] for the prenatal only, 3.50[2.51–4.90] for the postnatal only, 5.36[4.05–7.10] for the both).

**Table 2:** Multivariable logistic regression analysis for maternal psychological distress and behavioral problems in children

	Maternal psychological distress							
	None		Prenatal only		Postnatal only		Both	
	Crude OR (95% CI)	Adjusted OR (95% CI)^a^	Crude OR (95% CI)	Adjusted OR (95% CI)^a^	Crude OR (95% CI)	Adjusted OR (95% CI)^a^	Crude OR (95% CI)	Adjusted OR (95% CI)^a^
Externalizing problem	Ref		1.60 (1.29–1.97)	1.55 (1.25–1.92)	2.66 (2.14–3.29)	2.60 (2.09–3.24)	3.37 (2.80–4.05)	3.17 (2.63–3.83)
Internalizing problem	Ref		2.15 (1.77–2.61)	2.06 (1.69–2.52)	2.74 (2.21–3.38)	2.68 (2.16–3.34)	3.94 (3.30–4.71)	3.80 (3.17–4.57)
Total problem	Ref		2.57 (1.86–3.54)	2.39 (1.73–3.31)	3.75 (2.68–5.20)	3.50 (2.51–4.90)	5.89 (4.48–7.70)	5.36 (4.05–7.10)

*95% CI* 95% confidence interval, *OR* Odds ratio

^a^Adjusted for maternal age at delivery, parity, educational attainment, household income, maternal alcohol intake, maternal cigarette smoking, paternal cigarette smoking, child's sex

### Stratified analysis by the use of childcare facilities

Table 3 presents the ORs, 95% CIs, and *p*-values of the data on the interaction between the four categories of maternal psychological distress in early pregnancy and at two years postpartum, and behavioral problems in children aged four years through multivariate logistic regression analysis stratified by the use or no use of childcare facilities. The association of maternal psychological distress and high risk of children's behavioral problems was significant, except that a significant association was not identified between the prenatal only psychological distress and externalizing problems in the group that did not use childcare facilities. The footnotes to Table 3 mention the *p*-value for the interaction between the use of childcare facilities and the four categories of maternal psychological distress, with respect to the behavioral problems in children. As a result, the interaction was not significant at the 5% level of significance. *P*-value for interaction was respectively as follows; externalizing problems (0.200 for prenatal only, 0.280 for postnatal only, 0.634 for both), internalizing problems (0.976 for prenatal only, 0.064 for postnatal only, 0.210 for both), total problems (0.466 for prenatal only, 0.366 for postnatal only, 0.610 for both).

**Table 3:** Stratified analysis by the use of childcare facilities

	Use of childcare facilities (*n* = 2,924)							
	None		Prenatal only		Postnatal only		Both	
	Crude OR (95% CI)	Adjusted OR (95% CI)^a^	Crude OR (95% CI)	Adjusted OR (95% CI)^a^	Crude OR (95% CI)	Adjusted OR (95% CI)^a^	Crude OR (95% CI)	Adjusted OR (95% CI)^a^
Externalizing problem	Ref		1.86 (1.37–2.51)	1.84 (1.35–2.50)	2.25 (1.61–3.11)	2.31 (1.65–3.22)	3.57 (2.74–4.66)	3.47 (2.64–4.57)
Internalizing problem	Ref		2.16 (1.61–2.89)	2.03 (1.50–2.74)	2.1 (1.49–2.91)	2.08 (1.48–2.93)	3.47 (2.66–4.52)	3.31 (2.51–4.36)
Total problem	Ref		2.93 (1.82–4.67)	2.83 (1.76–4.55)	3.01 (1.77–5.01)	2.97 (1.76–5.00)	5.35 (3.57–8.09)	5.22 (3.43–7.95)
	**No use of childcare facilities (*n* = 3,335)**							
Externalizing problem	Ref		1.40 (1.04–1.87)	1.33 (0.99–1.79)	3.03 (2.26–4.03)	2.83 (2.11–3.80)	3.19 (2.74–4.66)	2.91 (2.64–4.57)
Internalizing problem	Ref		2.13 (1.64–2.75)	2.07 (1.59–2.70)	3.33 (2.51–4.38)	3.18 (2.39–4.22)	4.39 (3.46–5.58)	4.27 (3.34–5.46)
Total problem	Ref		2.30 (1.46–3.57)	2.04 (1.30–3.20)	4.39 (2.83–6.76)	3.91 (2.51–6.08)	6.38 (3.73–7.97)	5.45 (3.73–7.97)
	***P*-value for interaction**							
Externalizing problem	Ref		0.200		0.280		0.634	
Internalizing problem	Ref		0.976		0.064		0.210	
Total problem	Ref		0.466		0.366		0.610	

*95% *CI 95% confidence interval, *OR *Odds ratio

^a^Adjusted for maternal age at delivery, parity, educational attainment, household income, maternal alcohol intake, maternal cigarette smoking, paternal cigarette smoking, child's sex

## Discussion

In this large-scale birth cohort study in Japan, we examined whether the use of childcare facilities moderates the association of maternal psychological distress in the prenatal and postnatal periods with behavioral problems in children aged four years. The use of childcare facilities did not moderate the association between maternal psychological distress in the prenatal and postnatal periods and children's behavioral problems.

We first checked the association of maternal psychological distress in the prenatal and postnatal periods with behavioral problems in children. The psychological distress categories of prenatal only, postnatal only, and both were all associated with a high risk of externalizing problems, internalizing problems, and total problems, using the “none” category as a reference. These results are similar to findings of previous studies that reported the prenatal and postnatal maternal psychiatric symptoms to be associated with behavioral problems in children [4–14]. In particular, prenatal mental health has been consistently shown to be associated with children's development among various ethnic groups [4, 5, 9, 10]. We have confirmed this association in Japan. In the prenatal period, fetal in utero exposure to cortisol may cause reprogramming of the fetal hypothalamic–pituitary–adrenal (HPA) axis, leading to adverse developmental effects in children [18, 19]. Normally, 11β-hydroxysteroid dehydrogenase type 2 (11β-HSD2) in the placenta protects the fetus from cortisol exposure by inactivating cortisol. However, prior studies have shown that maternal anxiety is associated with the downregulation of placental 11β-HSD2 [18, 19, 37]. These findings indicate that maternal anxiety in early pregnancy is related to the exposure of the fetus to high levels of cortisol in utero through the down-regulation of 11β-HSD2 in the placenta. Thus, maternal psychological distress in early pregnancy is a prime candidate as a cause of fetal exposure to high levels of cortisol. In the postnatal period, a primary mechanism by which maternal psychological distress affects behavioral problems in children may be a decrease in the quality of parenting.

The use of childcare facilities did not moderate the association between maternal psychological distress in the prenatal period and children's behavioral problems in this study. One possible reason that the use of childcare facilities did not moderate the association could be that the use of childcare facilities does not affect children whose central nervous system and stress response system development was affected in utero. It has been shown that prenatal psychiatric symptoms may lead to reprogramming of the fetal HPA axis function [18, 19, 37]. Therefore, in order to prevent exposure to maternal psychiatric symptoms during the fetal period, it is important to prevent and cure maternal psychological distress in the prenatal period. Moreover, since there are various factors associated with psychological distress in early pregnancy, it may be important to identify and carry out interventions in more high-risk pregnant women. For example, a low socioeconomic status and lack of social support, including partner support, have been reported to be risk factors for perinatal mental disorders [17].

In this study, the use of childcare facilities did not moderate the association between psychological distress and behavioral problems in children during the prenatal period as well as the postnatal period. Those results were inconsistent with prior study results that showed that the use of childcare facilities moderates the association between postpartum depressive symptoms and behavioral problems in children [11, 15, 16]. The cause is unknown; however, it is possible that the perinatal and child-rearing environments differ in the Western and Asian countries [38, 39]. In fact, prior studies on this topic have been conducted in Canada, Australia, and the United States [11, 15, 16]. In Asia, common factors that affect perinatal depression have been reported as conflicts with work-life balance, poor relationships with the biological mother or in-laws, premarital pregnancy, and dissatisfaction with the infant's sex [38, 39]. Given these differences in the environmental factors, it is necessary to examine the mitigating factors specific to the Japanese population. In addition, the terms and conditions of use of childcare facilities may be a factor in why the use of childcare facilities did not alleviate the association between maternal psychological distress and children's behavioral problems. Most childcare facilities in Japan are used to taking care of children while their parents are working, and it is seldom permitted for unemployed mothers with psychological distress to use childcare facilities. Although mothers sometimes use childcare facilities because of difficulties in raising their children due to mental illness, the exposure in this study was psychological distress, and that severity is not enough reason to use a childcare facility. As mentioned above, mothers with psychological distress do not always have access to childcare facilities. If mothers experiencing psychological distress are more accessible to childcare facilities, the use of childcare facilities may be a factor in alleviating the association between maternal psychological distress and children's behavioral problems.

As far as we know, this is the first study to examine whether the use of childcare facilities can alleviate the association between psychological distress in the prenatal periods and behavioral problems in children; there are two implications in this study. First, this study suggests the need to identify and intervene mothers experiencing prenatal psychological distress at the earliest. The major interventions for maternal psychological distress are psychosocial support, such as life support, environmental adjustment, habit revision, and learning stress coping [40–44]. In the case of mothers diagnosed with depression or anxiety, psychotropic intervention may also be considered. Screening for psychological distress is also important to identify mothers who should be intervened with. Assessment of maternal mental health in the one-month postpartum period is widely conducted in Japan [45]. On the other hand, we propose focusing more on assessing maternal mental health during pregnancy and providing psychosocial support for mothers with psychological distress during pregnancy. It can potentially improve maternal psychological distress and the child's development. Second, this study implicates the need to identify the additional factors that may moderate the association between maternal psychological distress and behavioral problems in children. Since the moderating factors may differ among genetic and environmental factors, it is necessary to explore the moderating factors characteristic of the Japanese population.

There are a few limitations in this study. First, the TMM BirThree cohort study was conducted primarily in the Miyagi Prefecture, which limits the generalizability of the findings. Second, the TMM BirThree Cohort Study did not collect information on the type of childcare facilities, which could help us examine the ones beneficial for admitting the children. Third, since we focused on psychological distress as the exposure, our study is limited to comparisons of only the prior studies that examined whether the use of childcare moderates the association of maternal depression symptoms and behavioral problems in children. Fourth, since the CBCL is a parent-completed questionnaire, if the mother completing the CBCL has psychological distress, the child's behavioral problems may not have been correctly assessed. Fifth, the psychological distress may begin from the prenatal period and last until after delivery, leading the period of psychological distress to vary. Finally, the CBCL was only used for children aged four. Therefore, as the follow-up study progresses, it will be necessary to conduct the study when the participants are of an older age.

## Conclusion

This study showed an association of psychological distress in early pregnancy and at two years postpartum with a higher risk of behavioral problems in children aged four years. The association was strongest among mothers who experienced both prenatal and postnatal psychological distress. Moreover, the use of childcare facilities did not alleviate the association between maternal psychological distress and children's behavioral problems.

## Supplementary Information

**Additional file 1.**

## Data Availability

The data that support the findings of this study are available from the TMM biobank; however, restrictions apply to the availability of these data, which were used under license for the current study and hence are not publicly available. Data are available from the authors upon reasonable request and with the permission of the TMM biobank. All inquiries about access to the data should be sent to the TMM biobank (dist@megabank.tohoku.ac.jp).

## Abbreviations

K6: Kessler Psychological Distress Scale
CBCL: Child Behavior Checklist for Ages 1½–5
Cis: Confidence intervals
ORs: Odds ratios
TMM BirThree Cohort Study: Tohoku Medical Megabank Project Birth and Three-Generation Cohort Study
HPA axis: Hypothalamic-pituitary-adrenal axis
11β-HSD2: 11β-Hydroxysteroid dehydrogenase type 2
APA: American Psychological Association
